# Practical Notes and Formulæ

**Published:** 1888-03

**Authors:** 


### PRACTICAL NOTES AND FORMULÆ.

**Dr. P. Fedoroff**, of Arkhangelsk, states (*Proceedings of the Arkhangelsk Medical Society*) that he has obtained good results in whooping-cough by the internal use of the following mixture:

℞. Morphine, muriatic............................gr. ij,

Apomorphin, muriatic.................. .......gr. j,

Acid, muriatic...............................ʒ ss,

Aq. distill..................... ..............ʒ viij.

S.—A tablespoonful four times a day.

The paroxysms are lessened both in number and frequency after the first few doses of the mixture.—*The Pacific Record.*

**For Chronic Gonorrhœa and Gleet.**—Half a grain of corrosive sublimate and 4 grains of permanganate of potash, in 8 ounces of water, forms an excellent injection for chronic gonnorrhœa and obstinate gleet.—*Pacific Record.*

**Delirium of Typhoid Fever.**—Prof. N. S. Davis, Chicago:

℞. Strychniæ...................................gr. i,

Acidi nitrici.................................ʒ i,

Tr. opii......................................℥ iv,

Aquæ..........................................℥ iijss.

M. Sig.—A teaspoonful in sweetened water every two, three, four or six hours, according to the urgency of the symptoms.—*Ib.*

**Pyæmia.**—Professor S. D. Gross, Philadelphia:

℞. Quinn. sulph..................................gr. x,
Morph. sulph..................................gr. ½.

M. Sig.—This amount every four or six hours.—*Ib.*

**Acute Tonsilitis.**—Dr. O. S. Armstong, Detroit:

℞. Fl. ext. guaiac...............................ʒ ivss,

Syr. simp. q. s...............................℥ iv.

M. Sig.—Teaspoonful every hour in early stages.—*Ib.*

**Acute Rheumatism.**—Prof. J. E. Clark, Detroit:

℞. Acid salicylic...................... ......ʒ ivss,

Sodi bicarb...................................ʒ iij,

Aquæ..........................................q. s.

Miscet et adde:

Vini colchici.......................... ..........ʒ iss,

Elix. simp., q. s.............................℥ iv.

M. S.—Two teaspoonsful every two hours.—*Ib.*

